# Knowledge of the Therapeutic Goals of Diabetes Care among Patients with Type 2 Diabetes at a Tertiary Hospital in Ethiopia

**DOI:** 10.4314/ejhs.v32i3.15

**Published:** 2022-05

**Authors:** Melaku Taye, Kehabtimer Shiferaw, Paulos Efrem, Getahun Tarekegn

**Affiliations:** 1 Department of Internal Medicine, College of Health Sciences, Addis Ababa University, Addis Ababa, Ethiopia; 2 School of Public Health, College of Health Sciences, Addis Ababa University, Addis Ababa, Ethiopia

**Keywords:** diabetes mellitus, knowledge, therapeutic goals, triple targets

## Abstract

**Background:**

A comprehensive cardiovascular risk control reduces diabetes-associated complications but achieving the triplet goals (blood glucose, blood pressure (BP), and low-density lipoprotein (LDL-C)) remains a clinical challenge. Patients' knowledge of these diabetes therapeutic goals has been shown to improve glycemic control. However, this is not well studied in Ethiopia.

**Methods:**

A cross-sectional study was conducted from March to June 2020 in Tikur Anbessa Specialized Hospital among randomly selected 419 patients with type 2 diabetes. Data was collected using a pretested, structured questionnaire. A multivariable binary logistic regression was fitted to identify determinants of knowledge.

**Results:**

The mean age (±SD) of study participants was 57.4 (±10.8) years and the median duration (IQR) of diabetes was 12 (7, 20) years. A quarter of them achieved fasting glycemic control, a fifth of them attained the A1c goal and only 3% achieved the triple targets. Patients who reported knowing their target goals for BP, fasting blood sugar (FBS), A1C, and LDL-C were 79.5, 77.3, 11.7, and 7.2% respectively. The factors associated with knowledge of the therapeutic goals were longer diabetes duration, increased household income, age, being divorced as compared to married, having no formal education, and primary education.

**Conclusion:**

The majority of participants knew their BP and FBS targets while only a minority understood their A1c and LDL-C targets. It highlighted the need for the provision of patient-centered diabetes education to improve knowledge of the therapeutic targets.

## Introduction

Diabetes mellitus is a progressive metabolic disorder. It has become one of the fastest-rising global public health challenges with significant morbidity and mortality. Nearly half a billion people (9.3% of adults) are living with diabetes worldwide with over 4 million diabetes-related deaths annually. The majority of diabetes occurs in low and middle-income countries, imposing a significant economic burden (1).

Ethiopia is among the highest hit African countries. The estimated national prevalence of diabetes is 3.2% in adults of age 20–79 years (2). The pooled prevalence of diabetes in Addis Ababa is about 5% (3).

Despite the established benefits of integrated multiple risk factor reduction (including glycemic control, blood pressure optimization, and lipid-lowering therapy) (4,5), achieving these triplet goals remained a global clinical challenge. Achievement of these triple goals of diabetes care concurrently was successful in less than 10% of patients with type 2 diabetes (6–8).

Effective diabetes self-management and support are foundational for achieving the treatment goals of diabetes care. Patient-centered diabetes education to improve knowledge and self-care improves clinical outcomes and well-being cost-effectively (9).

It has been studied that patients' knowledge of the therapeutic goals of diabetes management improves self-care, medication adherence, and glycemic control. Lack of patients' knowledge of the therapeutic goals has been a strong predictor of poor compliance, suboptimal glycemic level, and inadequate control of other cardiovascular risk factors (10,11). Yet, there is a dearth of information on this topic in Ethiopia. Therefore, this study assessed patients' knowledge of the therapeutic goals of diabetes care that can be used as baseline information for further studies to implement appropriate interventions.

## Materials and Methods

An institution-based cross-sectional study was conducted at the endocrine clinic in Tikur Anbessa Specialized Hospital (TASH), Addis Ababa, Ethiopia. This clinic provides outpatient services to about 1000 patients with diabetes in a month. It was conducted from March to June 2020.

The study population was patients with type 2 diabetes attending the endocrine clinic in TASH for regular follow-up during the study period. All adult patients with type 2 diabetes with at least one prior visit to the clinic, and diabetes duration of 1 or more years since diagnosis were included.

Epi info version 7 was used to calculate sample size and determined as 422 using the formula to estimate a single population proportion. A simple random sampling technique was used to select the study participants. ODK version 1.25.2 software was used to collect the data along with the KoboToolbox server to store the collected data. Four BSc nurses who were trained on how to use this software collected the data.

Data on socio-demographic and behavioral factors were collected by interview using “WHO STEPS Instrument for Chronic Disease Risk Factor Surveillance” (12). To assess the study participants' knowledge of the therapeutic goals of diabetes, a 21-item knowledge questionnaire was administered. These items were adapted from prior studies in this area (10). Data on clinical and laboratory profiles of the participants were obtained through a review of digital medical records.

The therapeutic goals were measured as A1c ≤7%, FBS 80-130 mg/dl and BP <140/90 mmHg (5,13). The LDL-C goal was <100 mg/dl. Good knowledge was defined as knowing at least 5 out of 10 therapeutic target knowledge questions summed up.

The ODK collected data were validated and exported to SPSS 25 for analysis. Descriptive statistics included mean with SD and median with IQR for continuous variables, while frequency and percentage tables were used for categorical data.

Simple cross-tabulation and binary logistic regression analysis were used to study the association of independent variables with the knowledge of the therapeutic goals of diabetes management. Model fitness was checked using the Hosmer-Lemeshow goodness-of-fit test and was found to be fit. First, a bivariable analysis was done to identify the variables associated with the knowledge of the therapeutic goals of diabetes management. Then variables with P-value <0.25 in the bivariable analysis were selected as candidate variables and entered together into multivariable analysis to control for confounders. Lastly, variables with a p-value <0.05 in multivariable analysis were considered statistically significant and AOR with 95% CI was estimated to measure the strength of the associations. The result was presented by using text and tables.

**Ethical considerations**: Ethical approval was obtained from the departmental review board of Internal Medicine, College of Health Sciences, Addis Ababa University. Informed consent was obtained from each study participant. Data were analyzed anonymously.

## Results

**Demographic characteristics of the respondents**: A total of 419 patients participated in this study yielding a response rate of 99%. Two hundred sixteen participants (51.6%) were females. The mean age (±SD) was 57.4 (±10.8) years. The vast majority of the respondents (96.4%) were urban residents. About a third of the patients reported positive family history of diabetes (Table 1).

**Table 1:** Socio-demographic profile of patients with T2DM on follow-up at TASH, Addis Ababa, Ethiopia, 2020

Variable (n = 419)	Number (percent)
Sex	
Male	203 (48.4)
Female	216 (52.6)
Age category	
<35 years	2 (0.5)
35–44 years	55 (13.1)
45–54 years	109 (26.0)
55–64 years	137 (32.7)
.65 years	116 (27.7)
Residence	
Urban	404 (96.4)
Rural	15 (3.6)
Current marital status	
Single	16 (3.8)
Married	298 (71.1)
Divorced	44 (10.5)
Widowed	61 (14.6)
Education	
No education	28 (6.7)
Primary education	100 (23.9)
Secondary education	128 (30.5)
Higher education	163 (38.9)
Occupation	
Private worker	85 (20)
Government employee	79 (19)
Housewife	139 (33)
Retired	97 (23)
Others	19 (5)

**Clinical characteristics of the respondents**: The median duration of diabetes was 12 years, IQR (7, 20). Two hundred thirty-four (55.5%) of the participants had diabetes longer than 10 years duration. The median body mass index (BMI) was 25.6 with IQR (23.4, 28.6) kg/m^2^. Two hundred thirty-three (55.6%) of them have been diagnosed with hypertension. The majority of them, 303 (72.3%), reported that they attended diabetic education sessions. Only about half of them had their A1c and LDL-C checked in the last 1 year. Only 109 (26%) patients achieved the fasting glycemic target, and only 3% achieved the triple targets (Table 2).

**Table 2:** Clinical characteristics of patients with T2DM on follow-up at TASH, Addis Ababa, Ethiopia, 2020

Variables	Frequency (%)
Attended diabetic education (n = 419)	
Yes	303 (72.3)
No	116 (27.7)
Membership in diabetes association (n = 419)	
Yes	97 (23.2)
No	322 (76.8)
Own home glucometer (n = 419)	
Yes	284 (67.8)
No	135 (32.2)
Days glucose was measured per week (n = 419)	
None at all	89 (21.2)
Once or twice	227 (54.1)
Three times or more	103 (24.6)
Frequency of LDL-C checked in the last 1 year (n = 391)	
None at all	173 (44.1)
Once	101 (25.8)
Twice	76 (19.4)
Three-time or more	42 (10.7)
BMI (n = 419)	
Underweight	5 (1.2)
Normal	180 (43.0)
Overweight	162 (38.7)
Obese	72 (17.2)
A1c (n = 207)	
≤7%	48 (23.2)
>7%	159 (76.8)

**Patient-reported knowledge of the therapeutic goals of diabetes management**: The mean knowledge score was 4.7 ±2.2 points (out of 10). Two hundred fifty patients (59.7%) had a composite index of good knowledge of the therapeutic targets of blood pressure, FBS, A1c, and LDL-C. One hundred sixty-eight (40.1%) patients had ever heard about A1c testing. Knowledge about blood pressure and FBS targets was correct for 75.7% and 73% respectively while only 4.1% reported the correct target for A1c. Besides, knowledge about the target goal for LDL-C was poor as only 4 patients gave a correct answer (Table 3).

**Table 3:** Patient-reported knowledge of the therapeutic goals and current levels among patients with T2DM on follow-up at TASH, Addis Ababa, Ethiopia, 2020

Variable (n = 419)	Reports knowing target		Knows correct target		Knows own recent level	

	Number	Percent	Number	Percent	Number	Percent
BP (SBP or DBP)	333	79.5	317	75.7	303	72.3
FBS	324	77.3	306	73.0	336	80.2
A1c	49	11.7	17	4.1	70	16.7
LDL-C	30	7.2	4	1.0	34	8.1

**Determinants of knowledge of the therapeutic goals of diabetes management**: The result of the multivariable analysis identified age, marital status, educational status, average monthly household income, and duration of diabetes as independent determinants of knowledge status among people with type 2 diabetes.

For each one-year increase in the age of participants, the odds of having good knowledge of diabetes therapeutic goals decreased by 3% [AOR = 0.97; 95% CI: 0.95–0.99]. Besides, the odds of knowing the therapeutic goals for divorced patients with diabetes decreased by 63% as compared to married participants [AOR = 0.37%; 95% CI: 0.18–0.76]. Likewise, the odds of having a good knowledge score for participants with no education decreased by 97% compared to participants with above secondary education [AOR = 0.03; 95% CI: 0.006–0.13]. The odds of primary educated participants having good knowledge of therapeutic goals decreased by 73% [AOR = 0.27; 95% CI: 0.15–0.49] as compared to participants with higher education. As income increases by 1000 Ethiopian Birr (ETB), the odds of having good knowledge of therapeutic goals increased by 12% [AOR = 1.12; 95% CI: 1.02–1.23]. Besides, for each one-year increase in the duration of diabetes, the odds of having good knowledge increased by 4% [AOR = 1.04; 95% CI: 1.01–1.07] (Table 4).

**Table 4:** Bivariable and multivariable binary logistic regression analysis results of factors associated with knowledge status among patients with type 2 diabetes on follow-up at TASH, Addis Ababa, Ethiopia, 2020

Explanatory variable	Composite knowledge score		COR 95% CI	AOR 95% CI
			
	Good	Poor		
Age (years)	56.4±10.4€	58.9±11.4€	0.97 (0.96–0.99)	0.97 (0.95–0.99)*
Sex				
Male	137 (54.8)	66(39.1)	1.89(1.27–2.81)	1.19(0.59–2.43)
Female	113 (45.2)	103 (60.9)	1	1
Marital status				
Single	7 (2.8)	9 (5.3)	0.58(0.19–1.76)	0.40(0.13–1.26)
Married	193 (77.2)	105(62.1)	1	1
Divorced	15 (6.0)	29 (17.2)	0.38(0.17–0.86)	0.37(0.18–0.76)*
Widowed	35 (14.0)	26 (15.4)	1.37 (0.78–2.39)	1.59(0.80–3.13)
Occupation				
Private worker	61 (24.4)	24 (14.2)	1	1
Government employee	53 (21.2)	26 (15.4)	1.25 (0.64–2.43)	2.15(0.92–4.99)
Housewife	65 (26)	74 (43.8)	0.43 (0.24–0.77)	1.46(0.65–3.12)
Others	71 (28.4)	45 (26.6)	0.77(0.43–1.41)	1.49 (0.66–3.39)
Education				
No education	2 (0.8)	26 (15.4)	0.02(0.01–0.11)	0.29(0.01–0.13)*
Primary	42 (16.8)	58 (34.3)	0.23(0.13–0.39)	0.27(0.15–0.49)*
Secondary	82 (32.8)	46 (27.2)	0.56 (0.34–0.93)	0.65(0.37–1.13)
Higher	124 (49.6)	39(23.1)	1	1
Monthly income (ETB)	3000 (1600, 5000)¥	1900 (1000, 3000)¥	1.25(1.14–1.37)	1.12(1.02–1.23)*
Duration of diabetes (years)	14(8, 20)¥	10(5, 18)¥	1.02 (0.99–1.05)	1.04(1.01–1.07)*
Diabetes Education				
No	61 (24.4)	55 (32.5)	1	1
Yes	189 (75.6)	114(67.5)	1.49 (0.97–2.30)	1.38 (0.82–2.34)
Membership in DM association				
No	187 (74.8)	135 (79.9)	1	1
Yes	63 (25.2)	34(20.1)	0.75(0.47–1.19)	1.02 (0.58–1.77)
Have glucometer				
No	69 (27.6)	135 (79.9)	1	1
Yes	181 (72.4)	34(20.1)	1.68(1.11–2.54)	0.89 (0.50–1.56)
Glucose measurement				
None	40 (16)	49 (29.0)	1	1
Once weekly	72 (28.8)	53 (31.4)	1.66 (0.96–2.88)	1.38 (0.72–2.64)
Twice weekly	70 (28)	32 (18.9)	2.68 (1.48–4.84)	1.94 (0.94–3.98)
≥3 times weekly	68 (27.2)	35 (20.7)	2.38(1.33–4.27)	1.57(0.74–3.31)
Exercise adherence				
Non-adherent	73 (29.2)	67 (39.7)	1	1
Adherent	177 (70.8)	102 (60.3)	1.59(1.05–2.40)	1.19(0.73–1.95)

* Statistically significant at P-value ≤0.05

€ mean ±SD

¥ median with (Q1, Q3)

## Discussion

This was the first comprehensive assessment of the knowledge of the three therapeutic goals of diabetes care and its determinant factors amongst type 2 diabetes patients in Ethiopia. It showed that the proportion of patients with a good composite knowledge score was 59.7% with a mean knowledge score of 4.7 ±2.2 points (out of 10).

The patient population analyzed was representative of the patients with diabetes following at TASH. The mean age, sex, duration of diabetes, educational status, and place of residence in prior studies conducted in the TASH diabetes clinic was similar (13,14). The fasting glycemic control level in this study was also similar to a recent survey looking at the achievement of diabetes treatment goals in TASH (16). However, the percentage of patients taking insulin and practice of weekly blood glucose measurements was higher in this study, probably due to the higher likelihood of clinic visits by patients taking insulin than those on oral drugs who were being managed virtually during the beginning of the COVID-19 pandemic.

On the other hand, knowledge of fasting glycemia targets was better than in a previous study in TASH (15). The percentage of patients knowing their FBS target was also higher than that reported by an institution-based cross-sectional survey done in Dessie, Ethiopia (17). The differences in educational level and urban residency could contribute to this significant disparity in the knowledge of the FBS target.

Lipid profile measurement and statin use were higher than in previous studies in TASH and Jimma (15,18). Likewise, HbA1c determination is becoming an increasingly utilized tool for glycemic monitoring compared to previous studies in TASH (15,19). This is an encouraging finding indicating successful efforts of care providers availing such laboratory services in public health institutions to improve diabetes care.

Overall, patients' knowledge of BP and FBS targets and their recent levels were far better than that for A1c and LDL-C. These are much in line with those reported by a study conducted in South Carolina and China where the knowledge of the A1c and LDL-C goals was reported by less than one-third of patients while the rest majority were unknowing of their therapy targets (20–22). The higher familiarity of the respondents with BP and FBS targets and their recent values could be attributed to the fact that these measurements are commonly performed at each clinic visit compared to A1c and LDL-C tests which are less frequently determined. The higher cost and less availability of A1c and LDL-C testing could also contribute to a very low understanding of these tests while automated BP cuffs and glucometers are more accessible.

The multivariable analysis of this study identified five factors as independent determinants of knowledge of therapeutic goals among patients with type 2 diabetes. Good knowledge was found in younger patients, higher educational status, and married individuals. Likewise, increasing monthly household income and a longer duration of diabetes are statistically associated with good knowledge.

The relationship between increasing age and low educational level with poor knowledge was in agreement with studies from China (21,23), Kuwait (24), and Saudia Arabia (25). Age was found to be negatively correlated with diabetes knowledge in these studies. Older patients scored lower in diabetes knowledge. It could be due to increased cognitive impairment and memory loss with increasing age. Besides, patients with higher education tend to have better access to sources of knowledge about diabetes care with fewer barriers to communicating with diabetes educators.

Similar to our study, other researchers have reported better knowledge with a longer duration of diabetes but not with income and marital status (25–28). However, these studies used different instruments that assessed general knowledge about diabetes, not specific to the therapeutic goals. The possible explanation is that patients with a longer duration of diabetes tend to obtain a significant amount of diabetes-related knowledge over the years.

Attending diabetes education sessions, being a member of the diabetes association, and having a positive family history of diabetes were not associated with good knowledge of the therapeutic goals of diabetes in this study. In western studies, however, these factors were associated with overall diabetes knowledge status (29–32). Attending diabetes education has also been shown to be positively associated with good diabetes-related knowledge in Ethiopia (28,29). Lack of informing patients clearly about their goals of care during diabetes education sessions could be a possible reason but it needs further assessment.

In comparison to several previous studies done in Ethiopia that evaluated different aspects of diabetes-related knowledge and self-care activities (14,17,33,35), this study confirmed a relevant knowledge deficit among patients with type 2 diabetes about the most relevant, proximate triple markers of diabetes management, namely blood glucose, blood pressure, and blood lipids.

The main limitations of the study are the predominance of participants with higher education from urban areas that may overestimate the knowledge status, the low health literacy level often making understanding A1c and LDL-C difficult, and being a single-centered study.

In conclusion, the majority of participants reported knowing their BP and FBS targets while only a minority of patients understood their A1c and LDL-C values and goals. Overall, the composite knowledge status about the triple therapeutic goals of diabetes care among patients with type 2 diabetes was suboptimal. Age, educational status, marital status, household income, and duration of diabetes were identified as independent determinants of knowledge status. The findings of this study may help to improve diabetes education by targeting the knowledge of the therapeutic goals as achieving goals of diabetes care is difficult without patients knowing these specific targets.
